# Investigating the impact of fungicides and mungbean genotypes on the management of pod rot disease caused by *Fusarium equiseti* and *Fusarium chlamydosporum*

**DOI:** 10.3389/fpls.2023.1164245

**Published:** 2023-05-10

**Authors:** Harwinder Singh Buttar, Amarjit Singh, Asmita Sirari, Anupam, Komalpreet Kaur, Abhishek Kumar, Milan Kumar Lal, Rahul Kumar Tiwari, Ravinder Kumar

**Affiliations:** ^1^Department of Plant Pathology, Punjab Agricultural University, Ludhiana, India; ^2^Department of Plant Breeding and Genetics, Punjab Agricultural University, Ludhiana, India; ^3^Department of Chemistry, Punjab Agricultural University, Ludhiana, India; ^4^Department of Plant Pathology, Chaudhary Charan Singh Haryana Agricultural University, Hisar, India; ^5^Department of Plant Protection; Department of Crop Physiology, Biochemistry & Postharvest Technology, Indian Council of Agricultural Research (ICAR)-Central Potato Research Institute, Shimla, Himachal Pradesh, India

**Keywords:** propiconazole, screening, resistance, tabuconazole, trifloxystrobin

## Abstract

**Introduction:**

Mungbean is a vital pulse crop in India that can thrive in dry-land conditions and is grown in three seasons, with the added benefit of being used as green manure due to its ability to fix atmospheric nitrogen. Recently, pod rot disease has emerged as a serious threat to mungbean cultivation in India.

**Methods:**

In this study, morpho-molecular identification of associated pathogens and the bio-efficacy of systemic and non-systemic fungicides as well as genotype screening was performed during the years 2019 and 2020. The pathogens associated with this disease were confirmed on the basis of morphological and molecular characterization. For the molecular characterization, the translation elongation factor 1-alpha (tef-1) gene sequences were amplified by using primers (EF1 and EF2).

**Results:**

Under in vitro conditions, trifloxystrobin + tebuconazole 75% WG was found to be the most effective against Fusarium equiseti (ED_50_ 2.39 μg ml^−1^) and Fusarium chlamydosporum (ED_50_ 4.23 μg ml^−1^) causal agents of pod rot of mungbean. Under field conditions, three applications of trifloxystrobin + tebuconazole 75% WG at 0.07% as a foliar application at fortnightly intervals starting from the last week of July proved to be the most effective against pod rot disease on mungbean cultivars, i.e., ML 2056 and SML 668. To identify the potential resistance sources, 75 interspecific derivative and mutant lines of mungbean were screened for disease reaction to pod rot under natural epiphytotic conditions for the years 2019 and 2020. Genotypic differences were observed for resistance to pod rot disease. The study revealed that among the tested genotypes, ML 2524 exhibited resistance to pod rot disease, with a disease incidence of 15.62% and disease severity of 7.69%. In addition, 41 other genotypes were found to be moderately resistant (MR) to the disease.

**Conclusion:**

Altogether, the identified management options will offer an immediate solution to manage this disease under recent outbreak conditions and pave a path for futuristic disease management using identified resistant sources in breeding programs.

## 1 Introduction

Mungbean, also known as *Moong*, or *Green gram*, is one of the most important pulse crops in India. It is a short-duration crop that can tolerate dry-land conditions and is cultivated in three different seasons, *viz*., *Kharif*, *Rabi*, and summer, and also used as green manuring as having the capacity to fix atmospheric nitrogen (Nadeem et al., 2004; Pataczek et al., 2018). India contributes the maximum production of mungbean in South Asia, which accounts for around 90% of world output (Nair et al., 2012). The potential yield of mungbean is 2.5–3.0 t per hectare with selected varieties and good management practices under Indian conditions. However, the yield of mungbean in India is still relatively low, coming in at an average of 0.5 t per hectare (Nair et al., 2019). This could be because of changing climate conditions such as temperature and carbon dioxide levels within rain-fed areas, which can lead to varying levels of biotic stress (Chakraborty et al., 2000; Sharma et al., 2007). One of the most significant factors that contribute to this biotic stress is the presence of fungal infections, which can diminish the yield of mungbean by anywhere between 40% and 60% (Kaur et al., 2011). The loss of mungbean output that is caused by fungal diseases is a big worry for both the country’s farmers and the nation’s overall food security. Powdery mildew, rust, and anthracnose are just a few of the many fungal diseases that are frequent in the cultivation of mungbeans and are responsible for major losses in production. *Fusarium solani* and its related species are a significant concern among the various root rot pathogens and pose a considerable threat to several legumes, such as bean, pea, chickpea, and lentil (Carvalho et al., 2015; Naseri and Hamadani 2017a, Naseri, 2019). Additionally, *Fusarium virguliforme* (formerly known as *F. solani* f. sp. *glycine*) and other related *Fusarium* species are responsible for causing sudden death syndrome, which is a major issue for soybean cultivation in many regions (Rubiales et al., 2015).

Among the sustainable management approaches, agronomic and soil management practices have been reported highly effective in managing root rot in legumes (Naseri and Hemmati, 2017b; Naseri et al., 2018). The better understanding of agronomic practices and soil factors with special emphasis on soil texture, microbial populations, date of planting, method of cultivation, preceding crop, and fertilizer application are essential in root and stem rot disease management in legumes (Naseri et al., 2018). The introduction of beneficial microorganisms in the rhizosphere has also been found effective in controlling root rot pathogens. *Trichoderma* fungal species are commonly used for biological control of *Fusarium* incited rots in legumes. For example, treatment of lentil seedlings with *Trichoderma hamatum* was found to reduce colonization of *Fusarium oxysporum* f. sp. *lentis*, whereas soil application of *Trichoderma harzianum* in common bean- and chickpea-growing areas was found to effectively reduce the infection rates of *Fusarium oxysporum* f. sp. *phaseoli* and *Fusarium oxysporum* f. sp. *ciceris*, respectively (El-Hassan et al., 2013; Carvalho et al., 2015). However, it is a consistent challenge to find a highly efficacious strain of bioagent, which can effectively manage the disease under field conditions. For a newly emerging disease, the immediate solution is to identify broad-spectrum fungicides that can minimize the consistent outbreak in the farmers’ field. Parallelly, the exploitation of host plant resistance from the perspective of developing resistant varieties is the suitable long-term management strategy to manage root and stem rot diseases in legumes. During the last few years, pod rot disease caused by *Fusarium equiseti* and *Fusarium chlamydosporum* has emerged as a significant bottleneck in mungbean production (Buttar et al., 2023). The disease is characterized by pod discoloration and rotting of pods and seeds and aggravates if high rainfall occurs during maturity (Buttar et al., 2023). Since pods are the economic part of the crop, the farmers therefore have to achieve efficient control of this disease because it negatively affects the yield and subsequently their income. The disease is characterized by the rot of mungbean pods and stems, resulting in the death of infected plants. This not only reduces the yield of mungbean but also affects the quality of the beans by causing discoloration, cracking, and shriveling. Because the disease can also be transmitted through contaminated seed, it is absolutely necessary for the production of mungbeans to make use of seed that is of excellent quality and free of disease. There is a great need to prospect for the optimal approach in the management of pod rot to ensure efficient control. The application of broad-spectrum fungicides, both as a seed treatment and as a foliar spray, was the conventional strategy for disease control in mungbean production (Pandey et al., 2018). When it comes to the management of fungal infections in mungbean, utilizing host resistance has been acknowledged as a method that is both cost-effective and environmentally beneficial (Yadav et al., 2014a). The use of disease-resistant cultivars in conjunction with other agronomic and soil management practices is the most effective method for preventing and treating fungal diseases in mungbean crops (Pandey et al., 2018). As a reliable and quickly effective method, farmers prefer resistant varieties and use synthetic chemical pesticides to control the disease. Being a recently reported problem with the potential to inflict grave losses and lack of desirable host resistance, fungicides, bioagents, and botanicals were evaluated both under *in vitro* and field conditions against the associated pathogens. Moreover, the elite germplasms derived from interspecific hybridization between mungbean and ricebean/urdbean were screened for pod rot resistance to identify the resistant genotypes for resistance breeding programs.

## 2 Material and methods

### 2.1 Confirmation of pathogens associated with a pod rot disease

#### 2.1.1 Isolation, purification, and identification of the fungus

Mungbean pods showing symptoms of pod rot were collected from mungbean fields (untreated plots) of Punjab Agriculture University, Ludhiana, Punjab, India. Initial identification of pathogens based on cultural and morphological characteristics was done to confirm the association of *F. equiseti* and *F. chalamydosporum* with this disease. Field samples were sent to the laboratory for additional evaluation and isolation. The pods were meticulously cleaned and sliced into small bits. The components were sanitized before being placed on water agar medium for incubation under normal conditions. The cultures were then transferred to potato dextrose agar and purified using a single method of spore isolation. For further research, the pure cultures were maintained on PDA and Spezieller Nährstoffarmer Agar at 25 ± 1°C in the incubator. The cultural and morphological characters of the isolated pathogens were compared with those described previously by Leslie and Summerell (2006), Wollenweber and Reinking (1935), and Booth (1971).

#### 2.1.2 Molecular characterization of the pathogens

The identification of the pathogens causing pod rot was also confirmed by sequencing the translation elongation factor 1-alpha (tef-1) gene sequences. The mycelial mat of isolated pathogens causing pod rot of mungbean was grown in potato dextrose broth at 25 ± 10°C for 6 days in a BOD incubator. The total genomic DNA was isolated from mycelial mats using (Doyle and Doyle, 1987) DNA extraction methods with some modifications (CTAB method for DNA extraction). The translation elongation factor 1-alpha (tef-1) gene region from genomic DNA of both the fungal pathogens from genomic DNA was amplified with EF primers (EF1 and EF2) (O’Donnell et al., 1998) in polymerase chain reaction (PCR). For molecular-based identification of the culture, the purified PCR amplicons were outsourced for sequencing to Biologia Research India Pvt. Ltd. The rDNA tef-1 sequences were edited using MEGA-X Sequence viewer software. The edited nucleotide sequences were subjected to blast in three different tools, i.e., the “nucleotide BLAST” tool of NCBI (http://blast.ncbi.nlm.nih.gov/Blast) and the Fusarium MLST tool of the MYCOBANK database (https://fusarium.mycobank.org/) for its closest homology with database available in these databases for its identification.

### 2.2 Test fungicides

Commercial samples of seven fungitoxicants, *viz*., copper oxychloride (Blitox 50% WP, Rallis India Limited, Mumbai), propineb (Antracol 70% WP, Bayer Crop Science Limited, Mumbai), mancozeb (Indofil 75% WP, Indofil Chemicals, Mumbai), carbendazim (Bavistin 50% WP, BASF India Ltd., Mumbai), propiconazole (Tilt 25% EC, Bayer Crop Science Limited, Mumbai), tebuconazole (Folicur 25.9% WP, Bayer Crop Science Limited, Mumbai), and trifloxystrobin + tebuconazole (Nativo 75% WG, Bayer Crop Science Limited, Mumbai), were freshly procured from the market to evaluate their efficacy under *in vitro* and field conditions against pod rot in mungbean.

### 2.3 *In vitro* bioassay of fungitoxicants

To assess effectiveness under lab conditions, a poisoned food assay (Nene and Thapliyal, 1993) was conducted on potato dextrose agar (PDA) medium. The three non-systemic fungitoxicants, i.e., copper oxychloride, propineb, and mancozeb, were tested at a series of concentrations, *viz*., 0, 10, 25, 50, 100, 200, and 500 µg/ml, whereas systemic fungitoxicants (propiconazole, trifloxystrobin + tebuconazole, tebuconazole, and carbendazim) were tested at concentrations 0, 5, 10, 25, 50, 75, 100, and 200 µg/ml. The needed amount of the test chemical was combined with 100 ml of sterile PDA medium, and the resulting solution was poured under aseptic conditions into Petri plates with a 90-mm diameter. Each Petri dish contained a 7-mm circular slice of actively growing fungus, and each concentration was reproduced four times. The control were the Petri dishes using PDA medium without a fungicide. The Petri dishes were incubated at a temperature of 25 1°C. When the growth in the control plate reached 90 mm, the radial growth of the pathogens was measured, and the percent inhibition in colony growth (Pi) was computed using a formula published by Vincent (1947).

### 2.4 Field trials

The two-season field trials for evaluation of fungicides against pod rot disease were conducted at Krishi Vigyan Kendra, Goniana Sri Muktsar Sahib, Punjab, during 2019–2020 and at the experimental field, Pulse section, Department of Plant Breeding and Genetics, Punjab Agricultural University, Ludhiana, Punjab, during 2021 in a randomized block design with three replications. The location for the experiment was selected based on the previous history of high disease intensity in the chosen field. Cultivars ‘ML 2056’ and ‘SML 668’ for the years 2019–2020 and ‘ML 2056’ for the year 2021 were sown in the third week of July as per the standard agronomic practices recommended by Punjab Agricultural University (Anonymous, 2019).

Treatments details: Eight treatments (T1: copper oxychloride 50% WP at 0.3%, T2: propineb 70% WP at 0.3%, T3: mancozeb 75% WP at 0.3%, T4: carbendazim 50% WP at 0.2%, T5: propiconazole 25% EC at 0.1%, T6: tebuconazole 25.9% EC at 0.1%, T7: trifloxystrobin + tebuconazole 75% WG at 0.07%) were applied with untreated control for the years 2019–2020, and 10 treatments (T1: trifloxystrobin + tebuconazole 75% WG at 0.05%, T2: trifloxystrobin + tebuconazole 75% WG at 0.07%, T3: trifloxystrobin + tebuconazole 75% WG at 0.09%, T4: tebuconazole 25.9% EC at 0.08%, T5: tebuconazole 25.9% EC at 0.1%, T6: tebuconazole 25.9% EC at 0.3%, T7: *Trichoderma harzianum* at 1.5%, T8: *Pseudomonas fluorescens* at 1.5%, and T9: neem extract at 5%) that were applied with untreated control for the year 2021 were evaluated for management of pod rot of mungbean. Treatments were given as a foliar application of different fungicides (water volume for spray 500 l/ha using a knapsack sprayer fitted with a hollow-cone nozzle), and the untreated control was sprayed with water. Three applications were done for each treatment at an interval of 15 days starting from the last week of July. The disease incidence and severity were recorded 10 days after the last application following a 0–5 scale as described by Singh (2021). The percent disease index (PDI) was calculated as per the formulae given by Wheeler (1969). Percent incidence and percent disease control (PDC) were calculated according to the following:


$$\text{Percent incidence}=\frac{\mathrm{Number of diseased plants}}{\mathrm{Total no. of plants assessed}}\times \;100$$

$$\mathrm{Percent disease control}=\frac{\mathrm{PDI in Untreated}\;–\;\mathrm{PDI in Treatment}}{\mathrm{PDI in Untreated}}\times \;100$$

### 2.5 Field evaluation of mungbean genotypes for resistance against pod rot disease

Seventy-five interspecific derivative and mutant lines of mungbean (**Table 1**) were screened for reaction to pod rot under natural epiphytotic conditions for the years 2019 and 2020. The experiment was conducted in the Experimental area of the Pulses section, Department of Plant Breeding and Genetics, Punjab Agricultural University, Ludhiana, India. The test entries were sown on 4-m rows with a 30 × 10 cm spacing with two replications. Disease incidence and severity were recorded for each entry.

**Table 1:** Interspecific derivative and mutant lines of mungbean screened against pod rot disease.

S. no.	Germplasm	S. no.	Germplasm	S. no.	Germplasm	S. no.	Germplasm
1	MML 2539	20	MML 2575	39	SML 2063	58	ML 2482
2	MML 2543	21	MML 2577	40	SML 2064	59	ML 2483
3	MML 2544	22	MML 2578	41	SML 2065	60	ML 2498
4	MML 2545	23	MML 2579	42	SML 2066	61	ML 2500
5	MML 2546	24	SML 2048	43	SML 2067	62	ML 2506
6	MML 2549	25	SML 2049	44	SML 2068	63	ML 2507
7	MML 2550	26	SML 2050	45	SML 2069	64	ML 2524
8	MML 2551	27	SML 2051	46	SML 2070	65	ML 2525
9	MML 2560	28	SML 2052	47	SML 2071	66	ML 2566
10	MML 2561	29	SML 2053	48	SML 2073	67	ML 2567
11	MML 2562	30	SML 2054	49	SML 1839	68	ML 2568
12	MML 2563	31	SML 2055	50	SML 2015	69	ML 2569
13	MML 2565	32	SML 2056	51	SML 2016	70	ML 2576
14	MML 2568	33	SML 2057	52	ML 2423	71	MML 2566
15	MML 2570	34	SML 2058	53	ML 2459	72	MML 2567
16	MML 2571	35	SML 2059	54	ML 2460	73	MML 2569
17	MML 2572	36	SML 2060	55	ML 2479	74	MML 2576
18	MML 2573	37	SML 2061	56	ML 2480	75	MML 2580
19	MML 2574	38	SML 2062	57	ML 2481		

Percent incidence was calculated according to the following:


$$\mathrm{Percent incidence}=\frac{\mathrm{Number of diseased plants}}{\mathrm{Total no of plants assessed}}\times \;100$$

To record the data on pod rot disease severity, 100 pods were selected at random across the field, following a 0–5 scale (Singh, 2021).

**Table:** 

**Score**	**Disease description**
0	Apparently healthy plant
1	0%–10% of pod infected
2	11%–25% of pod infected
3	26%–50% of pod infected
4	51%–75% of pod infected
5	>75% of pod infected

The categorization of host reaction (HI, R, MR, MS, S, and HS) was done as follows on the basis of the per cent disease index:

**Table:** 

**Disease severity (%)**	**Reaction**
0	Highly resistant (HI)
0–10	Resistant (R)
10.1–25	Moderately resistant (MR)
25.1–45	Moderately susceptible (MS)
45.1–70	Susceptible (S)
More than 70.1	Highly susceptible (HS)

### 2.6 Statistical analysis

The data on percent growth inhibition under *in vitro* trials were subjected to analysis of variance (ANOVA) for factorial completely randomized design (CRD) to test the significance of the differences at a 5% probability level. ED_50_ and ED_90_ were calculated by Probit analysis using statistical software SPSS 16.0. The field data on disease incidence and disease severity was subjected to statistical analysis by Duncan’s multiple-range test employing R-studio software. The phylogenetic tree for the screened mungbean genotypes was prepared by using R-studio software (RStudio Team, 2021).

## 3 Results

### 3.1 Confirmation of pathogens associated with a pod rot disease

#### 3.1.1 Isolation, purification, and identification of the fungus

**Figure 1:**
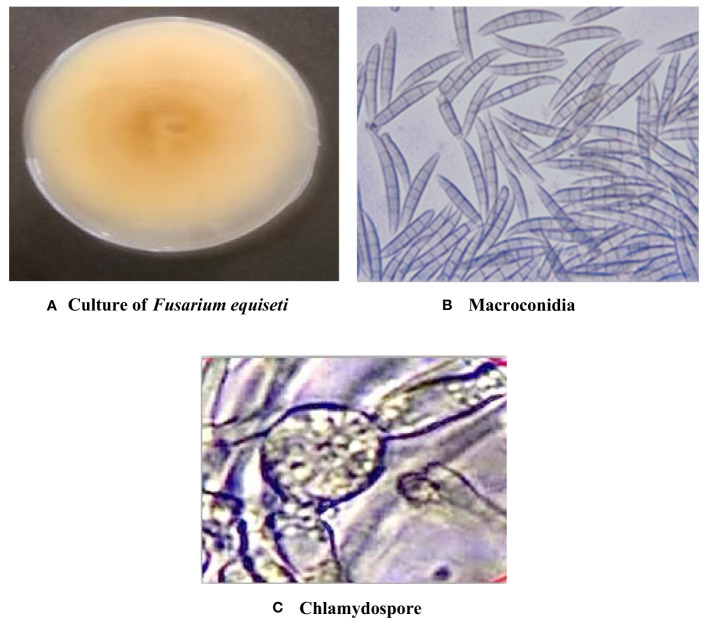
Cultural and morphological characteristics of the PR I isolate. **(A)** Colony morphology of *Fusarium equiseti.* **(B)** Macroconidia at 40× magnification. **(C)** Chlamydospores at 40X magnification.

**Figure 2:**
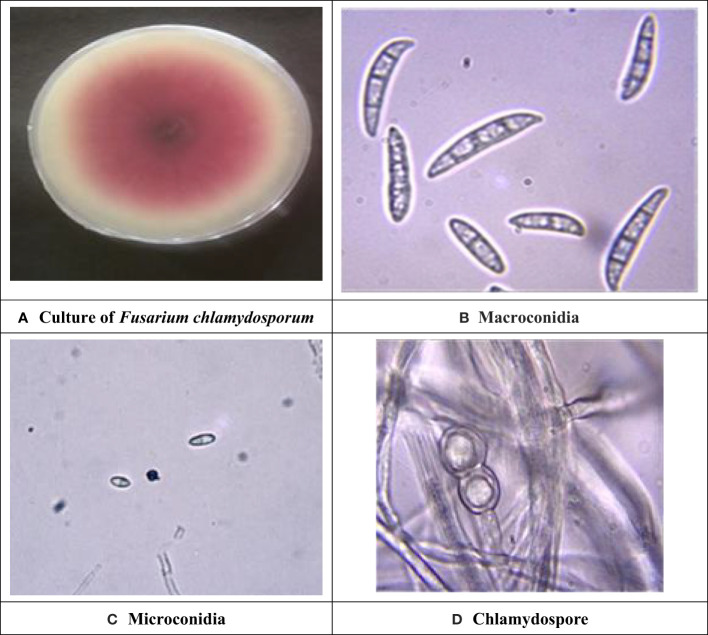
Cultural and morphological characteristics of PR II isolate. **(A)** Colony morphology of *Fusarium chlamydosporum.* **(B)** Macroconidia at 40× magnification. **(C)** Microconidia at 40× magnification. **(D)** Chlamydospores at 100× magnification.

Symptomatic mungbean pods exhibiting signs of pod rot were gathered from experimental plots in the mungbean fields at Punjab Agriculture University, located in Ludhiana, Punjab, India. Two isolates of Fusarium, i.e., PR I and PR II, were collected in this experiment. The cultures of both fungi were purified by employing the hyphal tip method (Tutte, 1969). The colony morphology of the PR I isolate (**Figure 1A**) was initially white with floccose abundant aerial mycelium changed to beige and finally to buff after 7 days with dense distribution, entire margins, and flat elevation. The conidia were falcate with a prominent foot cell and tapered and elongated apical cells with a pronounced dorsiventral curvature. Mature conidia usually contain five to seven septa measuring 28.42–52.32 × 3.8–5.9 µm (**Figure 1B**). Microconidia were not formed. Chlamydospores were formed singly or in chains with brown color, thick-walled, globose, and 5−10 μm in diameter (**Figure 1C**). Initially, the colony morphology of the PR II isolate (**Figure 2A**) was off-white with dark-pink pigments. Mycelium was floccose, snow-white initially, and later turned pink with irregular margins. After 7 to 14 days, dark-pink to burgundy pigmentation was observed in the culture, giving a dark-violet to black-colored appearance to cultures below at the agar base. The conidia were thick-walled, slightly curved from the upper side, and almost straight from the lower side with foot-shaped basal cells and pointed apical cells. Smaller conidia were aseptate to septate with two septa, and the larger ones usually contained three to five septa, measuring 22.72–38.62 × 2.7–6.1 µm (**Figure 2B**). Microconidia were oval to ovate, measuring 5.5–18.54 × 3.2–5.1 µm, and found in the aerial mycelium as being aseptate to having two septa (**Figure 2C**). Chlamydospores were abundant, verrucose with pale brown color, and formed singly or in chains (**Figure 2D**). The pathogens associated with pod rot of mungbean were confirmed PRI as *Fusarium equiseti* and PRII as *Fusarium chlamydosporum* after comparing the morphological features with those described previously by Leslie and Summerell (2006), Wollenweber and Reinking (1935), and Booth (1971).

#### 3.1.2 Molecular characterization of the pathogens

The pathogens were also identified by sequencing the translation elongation factor 1-alpha (tef-1) region, and nucleotide sequences were submitted to GenBank (NCBI). Results from the BLAST analysis of tef-1 sequences revealed that the PR-1 isolate was identified as *F. equiseti* (accession number: OK033107.1) based on the tef-1 gene sequence basis, whereas the PR-II isolate was identified as *F. chlamydosporum* (accession number: OK033106.1) (Buttar et al., 2023). In BLASTn search, sequences of isolate *F. equiseti* exhibited 96.49% resemblance with the sequences of *Fusarium equiseti* isolate PAK54 (https://blast.ncbi.nlm.nih.gov/Blast.cgi) and sequences of isolate *F. chlamydosporum* exhibited 97.07% resemblance with the sequences of *Fusarium chlamydosporum* strain DTO 418-C1 (https://blast.ncbi.nlm.nih.gov/Blast.cgi).

### 3.2 *In vitro* evaluation of non-systemic and systemic fungicides against *Fusarium equiseti* and *Fusarium chlamydosporum* causing pod rot of mungbean

**Figure 3:**
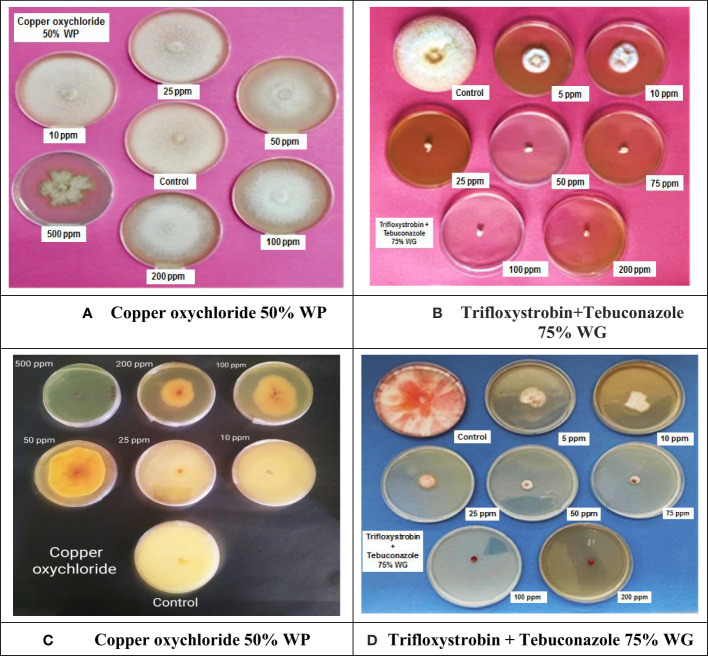
*In vitro* evaluation of non-systemic and systemic fungicides against *Fusarium equiseti* and *Fusarium chlamydosporum*: **(A)** Effectiveness of copper oxychloride 50% WP against *F. equiseti*. **(B)** Effectiveness of trifloxystrobin + tebuconazole 75% against *F. equiseti*. **(C)** Effectiveness of copper oxychloride 50% WP against *F. chlamydosporum*. **(D)** Effectiveness of trifloxystrobin + tebuconazole 75%against *F. chlamydosporum*.

All non-systemic fungitoxicants differed significantly at 10, 25, 50, 100, 200, and 500 µg ml^−1^ concentrations in terms of mean percent growth inhibition of *F. equiseti* (**Table 2**) and *F. chlamydosporum* (**Table 3**). Among the test non-systemic fungitoxicants, propineb 70% WP was the most superior among the non-systemic fungicides against *F. equiseti* with mean percent inhibition of colony growth of 35.63% and 41.88% mean growth inhibition of *F. chlamydosporum* followed by mancozeb 75% WP with 19.36% growth inhibition of *F. equiseti* and 27.60% growth inhibition of *F. chlamydosporum.* Copper oxychloride 50% WP proved to be the least effective in inhibiting the colony growth of both fungi (**Figures 3A, C**).

**Table 2:** *In vitro* evaluation of different fungicides against *Fusarium equiseti*.

Systematicity	Fungitoxicants	Percent growth inhibition over a check at different concentrations (µg ml^−1^)								
**Non-systemic**		5	10	25	50	75	100	200	500	Mean
	Copper oxychloride 50% WP	–	6.07 (14.23)	8.15 (16.39)	12.63 (20.55)	–	18.07 (25.13)	19.15 (25.93)	25.44 (30.27)	14.92 (22.08)
	Propineb 70% WP	–	25.70 (30.42)	30.63 (33.58)	31.56 (34.16)	–	35.44 (36.51)	40.52 (39.51)	49.93 (44.94)	35.63 (35.63)
	Mancozeb 75% WP	–	11.52 (19.57)	14.56 (22.21)	16.78 (24.16)	–	20.07 (26.60)	25.89 (30.56)	27.33 (31.48)	19.36 (25.76)
	**Mean**		14.43 (21.41)	17.78 (24.05)	2032 (26.29)		24.53 (29.41)	28.52 (31.99)	34.23 (35.56)	
**Systemic**	Carbendazim 50% WP	53.85 (47.19)	62.18 (52.64)	63.18 (52.63)	67.26 (55.12)	78.15 (62.10)	79.22 (63.13)	82.11 (64.99)	–	69.42 (56.75)
	Propiconazole 25% EC	49.63 (44.77)	53.59 (47.04)	61.04 (51.36)	62.41 (52.17)	71.78 (57.92)	72.85 (58.62)	77.30 (61.58)	–	64.08 (53.35)
	Tebuconazole 25.9% EC	57.30 (49.19)	62.44 (52.20)	68.81 (56.04)	72.37 (58.26)	78.81 (62.58)	79.52 (63.09)	83.51 (66.20)	–	71.83 (58.22)
	Trifloxystrobin + tebuconazole 75% WG	66.07 (54.37)	68.74 (56.01)	71.96 (58.01)	81.70 (64.69)	85.33 (67.50)	88.30 (69.98)	100.00 (89.65)	–	80.30 (65.75)
	**Mean**	56.71 (48.88)	61.74 (51.82)	66.25 (54.51)	70.93 (57.56)	78.51 (62.53)	79.97 (63.71)	85.73 (70.60)		

Non-systemic                Systemic:

CD (p=0.05)                 CD (p=0.05)

Fungitoxicants = 1.587             Fungitoxicants = 1.462

Concentrations = 2.244            Concentrations = 1.934

Fungitoxicants x Concentrations = NS      Fungitoxicants x Concentrations =3.868

**Table 3:** *In vitro* evaluation of different fungicides against *Fusarium chlamydosporum*.

Systematicity	Fungitoxicants	Percent growth inhibition over a check at different concentrations (µg ml^−1^)								
**Non-systemic**		5	10	25	50	75	100	200	500	Mean
	Copper oxychloride 50% WP	–	4.81 (12.60)	7.56 (15.93)	8.52 (16.94)	–	12.07 (20.32)	13.41 (21.39)	20.22 (26.69)	11.09 (18.99)
	Propineb 70% WP	–	30.52 (33.49)	34.96 (36.22)	41.52 (40.09	–	43.96 (41.51)	51.26 (45.70)	49.11 (44.47)	41.88 (40.25)
	Mancozeb 75% WP	–	12.74 (20.75)	16.44 (23.58)	19.33 (26.05)	–	24.96 (29.92)	39.81 (39.10)	52.33 (46.32)	27.60 (30.95)
	**Mean**	–	16.02 (22.28)	19.65 (25.24)	23.12 (27.69)	–	27.00 (30.58)	34.83 (35.39)	40.55 (39.16)	–
**Systemic**	Carbendazim 50% WP	54.19 (47.38)	63.41 (52.76)	67.59 (55.29)	71.00 (57.40)	75.96 (60.62)	79.56 (63.09)	83.33 (65.88)	–	70.72 (57.49
	Propiconazole 25% EC	53.56 (47.02)	62.41 (52.16)	69.22 (56.64)	72.44 (58.32)	77.96 (62.01)	83.33 (65.88)	100.00 (89.65)	–	74.20 (61.67)
	Tebuconazole 25.9% EC	72.44 (58.32)	74.15 (59.42)	79.44 (63.06)	82.22 (65.05)	89.00 (70.60)	100.00 (89.65)	100.00 (89.65)	–	85.32 (70.82)
	Trifloxystrobin + tebuconazole 75% WG	73.78 (59.17)	75.96 (60.63)	80.22 (63.60)	88.30 (69.97)	89.37 (70.94)	100.00 (89.65)	100.00 (89.65)	–	86.80 (71.95)
	**Mean**	63.49 (52.98)	68.98 (56.24)	74.24 (59.65)	78.49 (62.69)	83.07 (66.04)	90.72 (77.07)	95.83 (83.70)	–	

Non-systemic                 Systemic:

CD (p=0.05)                  CD (p=0.05)

Fungitoxicants = 1.628              Fungitoxicants = .846

Concentrations = 2.302             Concentrations = 1.119

Fungitoxicants x Concentrations = 3.988      Fungitoxicants x Concentrations =2.239

Among the test systemic fungicides trifloxystrobin + tebuconazole 75%, WG proved to be the most effective by providing 80.30% mean percent inhibition of *F. equiseti* colony growth (**Table 2**) (**Figure 3B**) and 86.80% mean percent inhibition of *F. chlamydosporum* colony growth (**Table 3**) (**Figure 3D**), whereas the tebuconazole 25.9% EC inhibits 71.83% colony growth of *F. equiseti*, which was at par with carbendazim 50% WP with 69.42% inhibition of colony growth. The least effective fungitoxicants among the systemic fungicides against *F. equiseti* was propiconazole 25% EC as it only recorded 64.08% colony growth inhibition and carbendazim 50% WP proved to be the least effective fungitoxicant against *F. chlamydosporum* 74.20% colony growth inhibition. At the concentration of 200 µg ml^−1^, trifloxystrobin + tebuconazole 75% WG proved to be the most effective as it completely inhibited the colony growth of *F. equiseti*, and at the concentration of 100 µg ml^−1^, trifloxystrobin + tebuconazole 75% WG and tebuconazole 25.9% EC proved to be the most effective against *F. chlamydosporum* as these completely inhibited the colony growth.

ED_50_ and ED_90_ values of different fungicides against *F. equiseti* (**Table 4**) and *F. chlamydosporum* (**Table 5**) were calculated and found that the propineb 70% WP had the lowest ED_50_ and ED_90_ values (429.22 and 963.62 µg ml^−1^) among the non-systemic fungicides tested against *F. equiseti* whereas mancozeb 75% WP and copper oxychloride 50% WP were less effective as the ED_50_ and ED_90_ values of these fungicides were 476.17 and 1166 µg ml^−1^ and 795.81 and 1524 µg ml^−1^, respectively. Among the systemic fungicides, trifloxystrobin + tebuconazole 75% WG recorded the lowest (2.40 µg ml^−1^) ED_50_ and ED_90_ (140.16 µg ml^−1^) values. ED_50_ (31.60 µg/ml) and ED_90_ (202.58 µg ml^−1^) values were recorded as the highest in the case of propiconazole 25% WP. ED_50_ and ED_90_ values of different fungicides against *F. chlamydosporum* are given in **Table 5**. Among the non-systemic fungicides tested against *F. chlamydosporum*, ED_50_ value 378.57 µg ml^−1^ was found to be the least value in the case of propineb 70% WP, whereas ED_90_ value 897.49 µg ml^−1^ was found to be the least value in the case of mancozeb 75% WP. Among the systemic fungicides, trifloxystrobin + tebuconazole 75% WG recorded the lowest (4.23 µg ml^−1^) ED_50_ and ED_90_ (54.78 µg ml^−1^) values. ED_50_ (18.51 µg/ml) and ED_90_ (102.59 µg ml^−1^) values were recorded as the highest in the case of propiconazole 25% WP.

**Table 4:** ED_50_ and ED_90_ values of different fungicides against pod rot of mungbean (*Fusarium equiseti*).

Fungicides	ED values (µg ml^−1^)	
	ED_50_	ED_90_
Non-systemic fungicides		
Copper oxychloride 50% WP	795.81	1524
Propineb 70% WP	429.22	1166
Mancozeb 75% WP	476.17	963.62
Systemic fungicides		
Carbendazim 50% WP	16.05	189.25
Propiconazole 25% EC	31.60	202.58
Tebuconazole 25.9% EC	3.86	211.88
Trifloxystrobin + tebuconazole 75% WG	2.39	106.16

**Table 5:** ED_50_ and ED_90_ values of different fungicides against pod rot of mungbean (*Fusarium chlamydosporum*).

Fungicides	ED values (µg ml^−1^)	
	ED_50_	ED_90_
Non-systemic fungicides		
Copper oxychloride 50% WP	962.02	1749.00
Propineb 70% WP	378.57	1252.00
Mancozeb 75% WP	417.39	897.49
Systemic fungicides		
Carbendazim 50% WP	11.80	184.24
Propiconazole 25% EC	18.51	102.59
Tebuconazole 25.9% EC	5.58	59.34
Trifloxystrobin + tebuconazole 75% WG	4.23	54.78

Copper oxychloride 50% WP was less effective as the ED_50_ and ED_90_ values of these fungicides were 981.69 and 1924.96 µg ml^−1^, respectively. Among the systemic fungicides, carbendazim 50% WP recorded the lowest ED_50_ (0.21 µg ml^−1^) and ED_90_ (76.32 µg ml^−1^) values. Propiconazole 25% EC was less effective as the ED_50_ and ED_90_ values of these fungicides were 3.64 and 247.69 µg ml^−1^, respectively.

### 3.3 Field evaluation of fungicides against pod rot of mungbean

All the treatments were found to be significantly different against pod rot disease in mungbean (**Table 6**). The minimum severity and maximum disease control of pod rot to the tune of 8.28% and 82.16%, respectively, were observed in treatment T7, i.e., trifloxystrobin + tebuconazole (75% WG) for SML 668, whereas it was 71.94% disease control with 10.94% severity for ML 2056. It was followed by T6, i.e., tebuconazole (25% EC) application showed 13.24% severity and 71.48% disease control for SML 668 and 13.21% severity and 66.11% disease control for ML 2056 over the control. However, T4 and T5 were adjudged at par statistically. Copper oxychloride (50% WP) was found to be the least effective among the evaluated fungicides with 25.16% disease control for ML 2056 and 36.54% disease control for SML 668. Application of propineb (70% WP) showed 39.72% and 33.25% disease control for SML 668 and ML 2056, respectively, which was adjudged at par with treatment T3 (mancozeb 75% WP).

**Table 6:** Field evaluation of different systemic and non-systemic fungicides against pod rot of mungbean during the years 2019–2020.

S. No.	Fungitoxicant	Dose (%)	ML 2056					SML 668				
			Incidence (%)	Severity (%)	PDC^*^	Yield (q/acre)	Yield loss (%)	Incidence (%)	Severity (%)	PDC^*^	Yield (q/acre)	Yield loss (%)
T1	Copper oxychloride (50% WP)	0.3	69.19^F^	29.17^B^	25.16	1.79^A^	61.09	71.42^C^	29.46^B^	36.54	2.13^A^	50.47
T2	Propineb (70% WP)	0.3	62.63^E^	26.02^B^	33.25	2.44^B^	46.96	70.68^C^	27.98^B^	39.72	2.47^B^	42.56
T3	Mancozeb (75% WP)	0.3	66.75^F^	27.75^B^	28.82	2.41^B^	47.61	68.84^C^	27.05^B^	41.73	2.39^B^	44.42
T4	Carbendazim (50%WP)	0.2	32.50^C^	15.92^A^	59.16	3.06^C^	33.48	26.32^B^	13.64^A^	70.62	3.09^C^	27.14
T5	Propiconazole (25% EC)	0.1	38.68^D^	18.17^A^	53.39	3.11^C^	32.39	28.64^B^	15.67^A^	66.24	3.13^C^	27.21
T6	Tebuconazole (25% EC)	0.1	27.00^B^	13.21^A^	66.11	3.59^D^	21.96	24.74^A^	13.24^A^	71.48	3.37^D^	21.63
T7	Trifloxystrobin + tebuconazole (75% WG)	0.07	22.57^A^	10.94^A^	71.94	3.66^D^	20.43	18.36^AB^	8.28^A^	82.16	3.57^D^	16.98
T8	Untreated control	–	79.37^G^	38.98^C^		1.66^A^	63.91	83.28^D^	46.42^C^		1.51^A^	64.88

*Percent disease control.

The values following the same letter are not significantly different according to Duncan’s multiple range test.

The minimum yield loss over control 16.98% was found for SML 668 when sprayed with trifloxystrobin + tebuconazole (75% WG), whereas it was 20.43% for ML 2056 in the same treatment. It was followed by 21.63% for SML 668 and 21.96% for ML 2056 in T6, i.e., tebuconazole (25% EC). Yield loss of 32.39% and 27.21% was observed in T5 for ML 2056 and SML 668, respectively, which was adjudged at par with treatment T4. Copper oxychloride (50% WP) was least effective, which showed 61.09% yield loss for ML 2056 and 50.47% yield loss for SML 668 followed by 47.61% for ML 2056 and 44.42% for SML 668 in T3, i.e., propineb (70% WP).

### 3.4 Field evaluation of different fungicides, bioagents, and neem extract against pod rot of mungbean during the year 2021

During the field evaluation of different fungicides against pod rot of mungbean in the years 2019–2020, trifloxystrobin + tebuconazole (75% WG) and tebuconazole (25% EC) were found to be effective against pod rot disease. Therefore, in the year 2021, these two fungicides were again evaluated against pod rot disease with different concentrations. In addition to these fungicides, two biocontrol agents, i.e., *Trichoderma harzianum* and *Pseudomonas fluorescence*, and one extract, i.e., neem extract, were also evaluated against pod rot disease of mungbean (cv. ML 2056) under field conditions. All the tested fungicides at different doses were found to significantly control the pod rot disease (**Table 7**). Among the tested fungicides, trifloxystrobin + tebuconazole (75% WG) at the rate of 0.09% was found most effective with 93.99% control of pod rot disease over control followed by trifloxystrobin + tebuconazole (75% WG) (91.48% PDC) at the rate 0.07% dose. Tebuconazole (25% EC) at the rate of 0.08% was found least effective among the fungicides with 55.78% disease control. Two biocontrol agents, i.e., *Trichoderma harzianum* at 1.5% and *Pseudomonas fluorescence* at 1.5%, were found less effective against pod rot disease with only 7.28 and 3.55 PDC, respectively, and showed the non-significant results with untreated control. Neem extract at the rate of 5% (2.90 PDC) was also found to be non-significant in pod rot disease control.

**Table 7:** Field evaluation of different fungicides, bioagents, and neem extract against pod rot of mungbean during the year 2021.

S. no.	Fungitoxicant	Dose (%)	ML 2056				
			Incidence (%)	Severity (%)	PDC*	Yield (q/acre)	Yield loss (%)
T1	Trifloxystrobin + tebuconazole (75% WG)	0.05	17.12^E^	8.51^D^	75.35	3.90^B^	15.22
T2	Trifloxystrobin + tebuconazole (75% WG)	0.07	8.18^F^	2.94^E^	91.48	4.51^A^	1.88
T3	Trifloxystrobin + tebuconazole (75% WG)	0.09	6.20^F^	2.08^E^	93.99	4.55^A^	1.09
T4	Tebuconazole (25% EC)	0.08	31.31^C^	15.27^B^	55.78	3.27^C^	28.99
T5	Tebuconazole (25% EC)	0.1	24.88^D^	11.98^C^	65.33	3.87^B^	15.94
T6	Tebuconazole (25% EC)	0.3	17.20^E^	8.36^D^	75.80	4.10^AB^	10.87
T7	*Trichoderma harzianum*	1.5	76.65^B^	32.03^A^	7.28	1.84^D^	60.00
T8	*Pseudomonas florescence*	1.5	79.19^AB^	33.31^A^	3.55	1.81^D^	60.58
T9	Neem extract	5	80.04^AB^	33.54^A^	2.90	1.69^D^	63.26
T10	Untreated control	–	82.87^A^	34.54^A^	0.00	1.67^D^	63.77

*Percent disease control. The values following the same letter are not significantly different according to Duncan’s multiple range test.

The yield of mungbean was found to be significantly changed with different fungicide treatments as compared with the untreated control, whereas the yield with biocontrol agents and neem extract was found to be statistically at par with untreated control. Minimum yield loss was supported by trifloxystrobin + tebuconazole (75% WG) (1.09%) at the rate of 0.09% dose followed by trifloxystrobin + tebuconazole (75% WG) (1.88%) at the rate 0.07% dose. While the yield loss with the treatment of trifloxystrobin + tebuconazole (75% WG) (15.22%) with 0.05% dose and tebuconazole (25% EC) (15.94%) at the rate of 0.1% were found statistically at par with each other.

### 3.5 Evaluations of genotypes for pod rot disease resistance

The data recorded revealed that all 75 genotypes of mungbean differed in their response to pod rot disease. These genotypes were categorized into six groups, i.e., highly resistant, resistant, moderately resistant, moderately susceptible, susceptible, and highly susceptible on the basis of disease severity (**Table 8**). The pod rot incidence of screened genotypes was found to be significantly different and ranged from 15.62% to 95.00% (**Table 9**). Of the tested genotypes, one genotype ML 2524 was found to exhibit resistant (R) reaction with 15.62% disease incidence and 7.69% disease severity. A total of 41 genotypes were found to be moderately resistant (MR) with disease incidence ranging from 23.37% to 58.39% and disease severity ranging from 11.38% to 24.91%. Twenty-four genotypes showed the moderately susceptible (MS) reaction having disease incidence of 60.52% to 85.19% and disease severity of 25.56% to 44.71% whereas nine genotypes, *viz*., MML 2543, MML 2545, MML 2551, MML 2560, MML 2563, MML 2575, ML 2566, ML 2568, and ML 2569, were found to exhibit susceptible (S) reactions with disease incidence that varied from 83.60% to 95.00% and disease.

**Table 8:** Reaction(s) of mungbean genotypes against pod rot disease.

S. no.	Disease severity (%)	Reaction	Genotypes
1	0	Highly resistant	–
2	0–10	Resistant	ML 2524
3	10.1–25	Moderately resistant	MML 2561, MML 2571, SML 2048, SML 2050, SML 2051, SML 2052, SML 2053, SML 2054, SML 2055, SML 2059, SML 2060, SML 2062, SML 2063, SML 2065, SML 2066, SML 2067, SML 2068, SML 2069, SML 2070, SML 2071, SML 2073, SML 1839, SML 2015, SML 2016, ML 2423, ML 2459, ML 2460, ML 2479, ML 2480, ML 2481, ML 2482, ML 2483, ML 2498, ML 2500, ML 2506, ML 2507, ML 2525, MML 2566, MML 2567, MML 2569, MML 2580
4	25.1–45	Moderately susceptible	MML 2539, MML 2544, MML 2546, MML 2549, MML 2550, MML 2562, MML 2565, MML 2568, MML 2570, MML 2572, MML 2573, MML 2574, MML 2577, MML 2578, MML 2579, SML 2049, SML 2056, SML 2057, SML 2058, SML 2061, SML 2064, ML 2567, ML 2576, MML 2576
5	45.1–70	Susceptible	MML 2543, MML 2545, MML 2551, MML 2560, MML 2563, MML 2575, ML 2566, ML 2568, ML 2569
6	More than 70.1	Highly susceptible	–

**Table 9:** Screening of mungbean interspecific derivates and mutant genotypes against pod rot disease.

S. no.	Genotypes	Incidence (%)		Pooled mean (%)	Severity (%)		Pooled mean (%)	Reaction
		2019	2020		2019	2020		
1	MML 2539	87.73	74.76	81.25	43.30	31.55	37.43	MS
2	MML 2543	80.73	86.46	83.60	40.55	49.68	45.12	S
3	MML 2544	76.05	67.48	71.76	38.80	28.05	33.43	MS
4	MML 2545	94.60	83.85	89.23	57.33	47.65	52.49	S
5	MML 2546	83.81	67.57	75.69	41.70	28.69	35.20	MS
6	MML 2549	91.22	73.48	82.35	51.09	30.93	41.01	MS
7	MML 2550	96.88	72.18	84.53	54.26	30.33	42.30	MS
8	MML 2551	100.00	87.43	93.72	55.56	43.44	49.50	S
9	MML 2560	79.35	97.83	88.59	42.02	54.80	48.41	S
10	MML 2561	51.40	40.00	45.70	22.38	18.52	20.45	MR
11	MML 2562	89.38	79.63	84.50	47.45	41.62	44.54	MS
12	MML 2563	88.32	83.41	85.86	49.95	42.43	46.19	S
13	MML 2565	95.45	72.81	84.13	59.12	30.29	44.71	MS
14	MML 2568	91.18	66.86	79.02	51.37	28.36	39.87	MS
15	MML 2570	91.25	68.46	79.86	50.70	28.86	39.78	MS
16	MML 2571	43.26	51.94	47.60	19.50	22.09	20.80	MR
17	MML 2572	85.29	67.71	76.50	47.93	28.22	38.08	MS
18	MML 2573	82.45	57.54	70.00	41.71	24.43	33.07	MS
19	MML 2574	60.91	66.03	63.47	32.82	27.28	30.05	MS
20	MML 2575	87.87	82.82	85.34	49.26	40.96	45.11	S
21	MML 2577	77.42	80.95	79.18	39.37	42.59	40.98	MS
22	MML 2578	66.28	74.54	70.41	36.17	31.44	33.81	MS
23	MML 2579	75.59	78.41	77.00	38.90	32.72	35.81	MS
24	SML 2048	60.33	54.26	57.30	25.67	23.80	24.73	MR
25	SML 2049	66.48	58.48	62.48	33.69	24.58	29.14	MS
26	SML 2050	51.64	51.41	51.53	22.20	22.45	22.32	MR
27	SML 2051	61.13	51.45	56.29	25.98	22.13	24.05	MR
28	SML 2052	56.25	39.51	47.88	23.78	18.01	20.89	MR
29	SML 2053	65.13	51.30	58.21	27.44	22.24	24.84	MR
30	SML 2054	57.81	44.79	51.30	24.22	19.14	21.68	MR
31	SML 2055	52.88	51.03	51.96	22.49	22.40	22.44	MR
32	SML 2056	82.33	70.99	76.66	40.13	30.33	35.23	MS
33	SML 2057	74.71	58.00	66.36	40.20	25.00	32.60	MS
34	SML 2058	61.77	59.28	60.52	26.11	25.01	25.56	MS
35	SML 2059	56.86	53.68	55.27	24.39	21.13	22.76	MR
36	SML 2060	48.29	46.41	47.35	20.97	20.08	20.53	MR
37	SML 2061	69.54	59.48	64.51	28.79	25.00	26.90	MS
38	SML 2062	48.48	50.82	49.65	21.22	22.29	21.75	MR
39	SML 2063	55.13	59.35	57.24	24.06	25.75	24.91	MR
40	SML 2064	75.71	47.87	61.79	39.06	20.70	29.88	MS
41	SML 2065	67.20	47.82	57.51	28.14	20.88	24.51	MR
42	SML 2066	52.27	52.03	52.15	22.98	22.52	22.75	MR
43	SML 2067	44.73	42.52	43.63	19.67	19.35	19.51	MR
44	SML 2068	40.53	44.12	42.33	18.40	18.93	18.67	MR
45	SML 2069	37.81	44.47	41.14	18.62	19.46	19.04	MR
46	SML 2070	20.10	26.64	23.37	9.80	12.96	11.38	MR
47	SML 2071	41.25	25.19	33.22	18.83	12.33	15.58	MR
48	SML 2073	40.57	31.64	36.10	18.60	15.68	17.14	MR
49	SML 1839	55.00	53.85	54.42	23.81	23.01	23.41	MR
50	SML 2015	58.14	46.81	52.47	24.85	20.00	22.42	MR
51	SML 2016	62.22	51.85	57.04	25.71	22.45	24.08	MR
52	ML 2423	50.00	45.71	47.86	21.37	19.88	20.62	MR
53	ML 2459	44.74	50.00	47.37	19.71	21.93	20.82	MR
54	ML 2460	42.86	27.27	35.06	18.71	13.37	16.04	MR
55	ML 2479	43.94	41.03	42.48	19.10	17.53	18.32	MR
56	ML 2480	22.64	40.48	31.56	11.10	17.91	14.50	MR
57	ML 2481	40.35	36.36	38.36	17.93	17.91	17.92	MR
58	ML 2482	43.94	40.00	41.97	19.02	19.14	19.08	MR
59	ML 2483	40.30	50.00	45.15	17.91	21.37	19.64	MR
60	ML 2498	46.34	55.77	51.06	19.97	24.25	22.11	MR
61	ML 2500	34.43	30.23	32.33	16.47	14.53	15.50	MR
62	ML 2506	43.14	58.33	50.74	18.84	25.47	22.16	MR
63	ML 2507	42.31	37.14	39.73	18.08	17.77	17.93	MR
64	ML 2524	7.55	23.68	15.62	3.77	11.61	7.69	R
65	ML 2525	24.14	25.71	24.93	11.89	12.79	12.34	MR
66	ML 2566	88.24	85.00	86.62	53.80	50.30	52.05	S
67	ML 2567	70.37	100.00	85.19	28.96	59.88	44.42	MS
68	ML 2568	90.00	100.00	95.00	50.85	56.50	53.67	S
69	ML 2569	80.65	85.19	82.92	45.05	49.53	47.29	S
70	ML 2576	70.45	87.10	78.78	29.85	55.83	42.84	MS
71	MML 2566	37.50	44.00	40.75	18.38	19.30	18.84	MR
72	MML 2567	35.59	46.88	41.23	17.03	20.20	18.62	MR
73	MML 2569	53.85	40.00	46.92	23.51	19.05	21.28	MR
74	MML 2576	100.00	63.33	81.67	54.35	26.39	40.37	MS
75	MML 2580	64.15	52.63	58.39	26.62	23.19	24.90	MR

## 4 Discussion

To manage root and stem rot diseases in legumes, it is essential to have a thorough understanding of agronomic practices and soil factors, including soil texture, microbial populations, planting date, cultivation method, preceding crop, fungicide requirements, and fertilizer application (Naseri and Hamadani, 2017a; Naseri et al., 2018). Moreover, the strategic application of fungicides to mitigate newly emerging plant disease has been believed to be a quick and effective control measure (Ahmad et al., 2021). It was concluded from the 2-year field trials of this study that pod rot disease in mungbean can be effectively controlled with the treatment of trifloxystrobin + tebuconazole (75% WG) and tebuconazole (25% EC) as a foliar application. Three applications at 15-day intervals starting from the last week of July countered the disease successfully. The active ingredient in each of these fungicides is tebuconazole, which is categorized as a demethylation inhibitor (DMI) fungicide. Inhibiting the cytochrome P450-dependent enzyme, DMI fungicides affect the manufacture of sterols, which are key components of fungal cell walls (Odds et al., 2003; Patón et al., 2017). Golakiya et al. (2018) reported tebuconazole + trifloxystrobin (73.50%) as the most effective among the six evaluated fungicides against Fusarium associated with the chickpea wilt. In a study conducted by Gabrekiristos and Ayana (2020), five fungicides were tested against the Fusarium wilt of hot pepper and found that Nativo SC 300 (trifloxystrobin + tebuconazole 100:200 g/l) and Twinstar 75 WG (trifloxystrobin + tebuconazole 50:25% w/w) fungicides have the nature of both systemic and contact action and led to 94.0% and 92.3% mycelia growth inhibition, respectively. Flint Max (tebuconazole 50% + trifloxystrobin 25%) was a very effective fungicide with an EC50 value of less than 1 ppm, at inhibiting mycelial growth of *F. proliferatum* causing bulb rot of garlic (Patón et al., 2017), and the results are consistent with earlier research on the efficacy of tebuconazole, the active ingredient in both Nativo and the fungicide evaluated in this study. Marin et al. (2013) determined that the EC50 (the concentration necessary to create 50% inhibition) and EC90 (the concentration required to produce 90% inhibition) of tebuconazole against *F. proliferatum* from wheat were 0.50 and 10.0 ppm, respectively. Ivić et al. (2011) determined the EC50 for tebuconazole to be between 0.85 and 2.57 ppm for *F. graminearum*, 0.85 and 1.58 ppm for *F. avenaceum*, and 0.22 and 0.85 ppm for *F. verticillioides*. *In vitro* testing by Jain et al. (2015) revealed that tebuconazole was effective against Fusarium associated with tomato, with IC50 values of 19.27 g ml^−1^ (the dose necessary to achieve 50% inhibition). DMI fungicides, such as tebuconazole, have been demonstrated to be more effective against Fusarium species than other fungicides. Based on EC50 and EC90 values, the triazole chemical group, which includes prothioconazole, cyproconazole, and tebuconazole, is more effective against Fusarium spp. than strobilurin fungicides (such as azoxystrobin and kresoxim-methyl) (Patron et al., 2016). Fusarium species are sensitive to fungicides belonging to the DMI group, such as tebuconazole, but innately resistant to complex III respiration inhibitors (QoI) such as trifloxystrobin, according to Dubos et al. (2011); Dubos et al. (2013) and Pasquali et al. (2013). Given these data, it appears that tebuconazole is responsible for the inhibitory effects seen in this investigation.

Prior to this study, there were no reports of sources of high pod rot disease resistance. The evaluation of mungbean germplasm for resistance to the pod rot pathogen allowed us to find one highly resistant genotype and 41 moderately resistant genotypes. In contrast to susceptible genotypes, these lines exhibited only moderate resistance, as evidenced by reduced symptoms and slower disease progression. Breeding programs may be able to increase the resistance levels of already existing cultivars with advantageous agronomic qualities by incorporating the resistance levels discovered in this study. This knowledge of resistance sources is advantageous for selecting germplasm for such programs (Larsen and Porter, 2010).

## 5 Conclusion

Mungbean is an important pulse crop that is widely grown for food, green manuring, and intercropping. It is of high economic importance due to its various uses and benefits. However, mungbean crops are susceptible to pod rot disease, which can cause significant yield losses if not managed properly. Pod rot disease can develop quickly under favorable weather conditions, especially in fields with a long history of mungbean cultivation, typically over 4–5 years. This disease can be devastating to crops and cause significant economic losses for farmers, and hence, chemical control and resistant varieties are valuable options for managing this devastating problem effectively. In conclusion, the management options identified in this study provide an effective means of managing pod rot disease in mungbean crops during recent outbreaks. Moreover, the identification of resistant sources through breeding programs can offer a sustainable long-term solution for managing the disease. Therefore, implementing these management options can provide an immediate solution to control the disease and pave the way for future disease management efforts.

## Data availability statement

The datasets presented in this study can be found in online repositories. The names of the repository/repositories and accession number(s) can be found in the article/supplementary material.

## Author contributions

HB, AmS: conceptualization, methodology, investigation, writing—original draft preparation. AsS, A, KK: methodology, software, visualization. RT, RK: reviewing and editing. AK, ML: supervision, project administration. All authors contributed to the article and approved the submitted version.
